# Notes and News

**Published:** 1892-11-12

**Authors:** 


### Notes and News.

COLLECTED AND COMMUNICATED.

Despite the depression and the fact that no equivalent for the Duke of Cleveland's £5,000 in 1891 was forthcoming this year, the results of the Metropolitan Hospital Sunday Fund are distinctly encouraging. The following table contains the figures for the years 1891-92. In 1891 the amount given under the head of legacies and donations, £4,020, is exclusive of the Duke of Cleveland's special gift:—

| | Contributing Congregations. | Amounts from Congregations. | Legacies and Special Donations. | Total. |
|---|---|---|---|---|
| 1891 | 1,711 | £36 311 | £4,020 | £40,331 |
| 1892 | 1,738 | 37,083 | 4,429 | 41,512 |

Thus in 1892 there was an increase of 27 in the number of contributing congregations, who paid into the fund £37,083, against £36,311 received in 1891. The total collections in 1892 amounted to £41,512, as against £40,331 in the previous year.

Those who are inclined to regard all progress in the present day as but a backward motion, and to apply too microscopic an eyeglass to the faults of our hospitals, should read and reflect on the account of the celebrated Hôtel Dieu, Paris, in past days, as described in the *Globe* of the 4th inst. Let it also be noted that "in these good old times" the institution was under that State management which some misguided individuals are inclined to consider as likely to be the panacea of all evils in hospital management. We learn that in 1781 Louis XVI. especially forbade the placing of more than two patients in one bed, which command, though tacitly permitting a condition of affairs incomprehensible to our enlightened ideas, was nevertheless a most necessary and beneficial alteration of that which had previously been the custom, for shortly before this three or four small-pox, several surgical, and other cases were placed in one bed, the beds in the first place being constructed to hold four persons. Later on beds of an improved pattern were supplied, to which sliding extension were added, and patients took it in turns to lie down, when the accommodation was entirely inadequate. It is to be wondered that patients could be found to enter the hospitals of those days, judging by the many exacting cases everywhere to be met with in present bright, clean, and comfortable institutions. The adage, "the more we have the more we want," holds very true in the hospital world as elsewhere; nevertheless, moments must come when the most active spirit of progress must pause, look back, and be thankful. The Hôtel Dieu is no bad subject for such contemplation.

We are naturally inclined to condemn a verdict, on our own judgment of a case, when insufficiently acquainted with the facts, but so apparently flagrant an injustice as that in the case of the girl of fourteen, recently fined £5 for selling poison at St. Helens, must surely afford some explanation not published in the account of the case. According to evidence, the girl was left in charge of the shop in the absence of her employer, and sold some oxalic acid to a man, who stated he required it for cleaning brass. Eventually he used it as a means of committing suicide, and thus the girl was charged with the sale, and fined, though unacquainted with the pharmacy laws. Her employer was held in no wise responsible. Surely all druggists should be liable if employing incompetent assistants. Otherwise, what security is offered to the public?

Those who glance at the report of the last quarterly meeting of the Radcliffe Infirmary, Oxford, will observe that although interest in the institution is practically dormant throughout the county and city, the authorities themselves have become convinced of the soundness of the views expressed by our Commissioner after a visit to the Infirmary more than a year ago. The Chairman, Dr. Bright, in the motion brought forward by him on the present occasion, drew up a list of requirements exceeding in length the number of objections raised by our Commissioner. He particularly dwelt on the necessity for better nurses' accommodation, and for wards better suited to the application of modern surgery. He designedly mentioned the full list of additions and improvements necessary to bring the institution up to the mark of efficiency, Lord Jersey having pointed out to him that he believed the appeals so far made had failed on account of a want of knowledge generally of what were the real needs of the Infirmary. Had the art of interesting the public in their hospital been better understood, we do not hesitate to affirm that the appeal for funds would have resulted in an infinitely larger sum than the insignificant £2,000 towards the £10,000 held to be necessary by our Commissioner. The object of the present quarterly meeting was to gain the consent of the governors to the use of two bequests, made some years ago for the purpose of structural improvements. These sums, together with the £1,800 subscribed, will amount to nearly £7,000 After some discussion the motion of the Chairman was carried, which provides that a sum of about £8,500 shall be devoted to structural improvements and additions. There was the usual display of timidity on the part of some governors present, which seems especially to characterise the meetings at the Radcliffe, but practical common sense and a faith in the success of a policy of progress and efficiency at last seems to have taken root in the management. We would again urge the Committee to call a public meeting, and to use every effort in their power to arouse general interest. This once done, we believe that the response would be large enough to prevent the proposed encroachment upon the invested funds.

